# Neuroinflammation Profiling of Brain Cytokines Following Repeated Blast Exposure

**DOI:** 10.3390/ijms241612564

**Published:** 2023-08-08

**Authors:** Lanier Heyburn, Andrew Batuure, Donna Wilder, Joseph Long, Venkatasivasai Sujith Sajja

**Affiliations:** Blast Induced Neurotrauma, Walter Reed Army Institute of Research, Silver Spring, MD 20910, USAandrew.b.batuure.ctr@mail.mil (A.B.);

**Keywords:** blast wave, cytokines, chemokines, neuroinflammation, brain

## Abstract

Due to use of explosive devices and heavy weapons systems in modern conflicts, the effect of BW on the brain and body is of increasing concern. These exposures have been commonly linked with neurodegenerative diseases and psychiatric disorders in veteran populations. A likely neurobiological link between exposure to blasts and the development of neurobehavioral disorders, such as depression and PTSD, could be neuroinflammation triggered by the blast wave. In this study, we exposed rats to single or repeated BW (up to four exposures—one per day) at varied intensities (13, 16, and 19 psi) to mimic the types of blast exposures that service members may experience in training and combat. We then measured a panel of neuroinflammatory markers in the brain tissue with a multiplex cytokine/chemokine assay to understand the pathophysiological process(es) associated with single and repeated blast exposures. We found that single and repeated blast exposures promoted neuroinflammatory changes in the brain that are similar to those characterized in several neurological disorders; these effects were most robust after 13 and 16 psi single and repeated blast exposures, and they exceeded those recorded after 19 psi repeated blast exposures. Tumor necrosis factor-alpha and IL-10 were changed by 13 and 16 psi single and repeated blast exposures. In conclusion, based upon the growing prominence of negative psychological health outcomes in veterans and soldiers with a history of blast exposures, identifying the molecular etiology of these disorders, such as blast-induced neuroinflammation, is necessary for rationally establishing countermeasures and treatment regimens.

## 1. Introduction

Traumatic brain injury (TBI) was one of the most common types of injury sustained during the conflicts in Iraq and Afghanistan, with more than 380,000 TBIs reported between 2000 and 2017, 82% of which were classified as mild (mTBI) [1]; mTBI is associated with psychiatric disorders, including post-traumatic stress disorder (PTSD) and depression [2,3], and military veterans studies indicate that TBI increases the risk of developing these psychiatric disorders among war veterans [4]. This risk may be further compounded by service members’ increased physical and psychological stress before and during combat deployment. A study of 2,441,698 active-duty service members between 2010 and 2015 found that ~18% were diagnosed with either TBI, PTSD, or chronic pain (together known as the polytrauma clinical triad (PCT)), and ~2% of individuals were diagnosed with both TBI and PTSD [5]. Due to the use of improvised explosive devices (IEDs) in modern conflicts, brain injury caused by primary blast (BW) exposure is of increasing concern for military populations. In addition, repeated low-intensity BW is commonly experienced during training and operations with breaching and heavy weapons systems. However, the manifestations and pathophysiological processes associated with these comorbidities are unclear. Several preclinical and clinical studies focused on the untoward effects of blast exposure have shown that ongoing inflammatory cascades in systemic organs and the brain could contribute to comorbidities [6,7,8].

Inflammation is a cellular and molecular response that is common across many types of injury and disease states. BW has been shown to induce neuroinflammation in the brain through glial activation and cytokine cascades [7,9,10]. Inflammation, with many cytokines and chemokines that are prominent contributors to pathophysiological processes, has been shown to be associated with psychiatric disorders, including PTSD and depression. For example, C-X-C motif chemokine ligand 2 (CXCL2) and CXCL3—chemokines that are primarily involved in neutrophil recruitment—are significantly decreased in depressed suicide victims compared to healthy controls [11]. Similarly, intercellular adhesion molecule-1 (ICAM-1), interleukin (IL)-1β, IL-6, IL-10, and tumor necrosis factor (TNF)-α are significantly increased in depression and bipolar disorder, while interferon (IFN)-γ is significantly decreased [12,13,14]. More specifically relevant to this study, brain injury and neuroinflammation are associated with psychological disorders in military service members. Cytokine production has been found to be an underlying mechanism of stress response in soldiers [15], with pro-inflammatory markers such as IL-1β, IL-6, and TNF-α being increased in PTSD patients [16]. The immune response is dysregulated in veterans with PTSD [17], and mTBI is associated with inflammation and pain [18], pointing to a similar underlying inflammatory mechanism within the polytrauma clinical trial population. Overall, TBI and associated comorbidities have been shown to have neuroinflammatory components that may be relevant to military-relevant BW symptomatology and pathogenesis.

Despite these apparent connections, the effects of commonly encountered low-level repeated blast exposures on these inflammatory cascades are unclear. Here, we investigate the acute neuroinflammatory profile resulting from repeated blast exposure in rats. We previously found that glial fibrillary acidic protein (GFAP), a marker of astrocyte activation involved in neuroinflammation, was increased in the brain following repeated low-intensity BW [19]. In order to investigate molecular neuroinflammation, we measured an array of pro- and anti-inflammatory cytokines and chemokines in the brain 24 h after BW exposure, with variables including blast intensity (13, 16, or 19 psi), blast frequency (1×, 2×, or 4×), and animal orientation (front-on or side-on), using an advanced blast simulator. We found that repeated low–moderate-intensity BW led to inflammatory changes associated with both TBI and psychological stress.

## 2. Results

A summary of the changes in neuroinflammatory markers using a multiplex assay is presented in Table 1. Overall, the animals with the most inflammatory changes were those exposed to multiple lower-intensity blasts. The 19 psi (front) exposure group did not have any significant changes, and the 19 psi (side) exposure group had changes in only two markers: the cytokine CXCL3 (47% increase over sham) and IL-10 (164% increase over sham). There were few other significant changes following just a single BW exposure. For 1× 16 psi (front) exposure animals, there were significant increases in CXCL3, ICAM-1, IL-2, IL-6, and IL-10, and for 1×-13 psi (side) exposure animals there was a significant increase in IL-10. No significant changes were observed after a single 16 psi exposure from the side or 13 psi exposure from the front. Two BW exposures led to increased brain levels of multiple inflammatory markers, but only in the 16 psi (side), 13 psi (front), and 13 psi (side) exposure groups. Similarly, four exposures led to altered brain inflammatory profiles, but not following a single BW exposure (Table 1).

### 2.1. Chemokine (C-X-C) Motif Ligands

**Figure 1:**
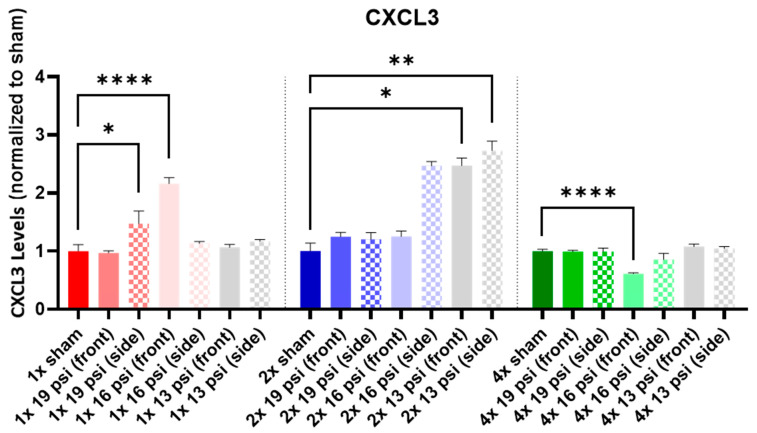
CXCL3 expression following repeated BW: CXCL3 was significantly increased following a single exposure to 19 psi (side) or 16 psi (front), two exposures to 13 psi (front and side), and significantly decreased following four exposures to 16 psi (front). Values are normalized to respective shams with the same number of exposures. Data are expressed as the mean ± SEM; * *p* < 0.05, ** *p* < 0.01, **** *p* < 0.0001.

**Figure 2:**
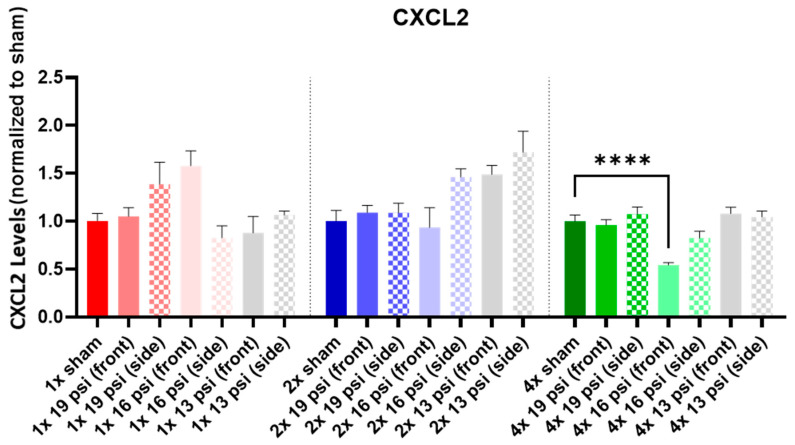
CXCL2 expression following repeated BW: CXCL2 was significantly decreased following four exposures to 16 psi from a front-facing orientation. Values are normalized to respective shams (1× = red, 2× = blue, 4× = green) with the same number of exposures. Data are expressed as the mean ± SEM; **** *p* < 0.0001.

CXCL3 was also significantly reduced (~39%) in the 4× 16 psi (front) exposure group (Figure 1). Conversely, CXCL3 was significantly increased following a single exposure to either 19 psi (side) or 16 psi (front) (~47% and ~116%, respectively). It was also increased following two exposures to 13 psi from the front (~147%) or side (~173%). CXCL2 was not significantly altered following either 1× or 2× BW exposures (Figure 2), although it was significantly reduced (~45%) following 4× 16 psi exposures from the front.

### 2.2. Intercellular Cell Adhesion Molecule 1 (ICAM-1)

**Figure 3:**
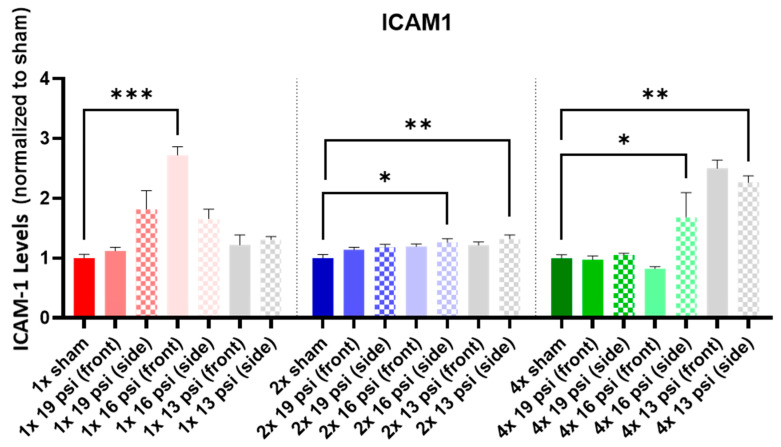
ICAM-1 expression following repeated BW: ICAM-1 was significantly increased following a single exposure to 16 psi (front), two exposures to 16 psi (side) and 13 psi (side), and four exposures to 16 psi (side) and 16 psi (front). Values are normalized to respective shams with the same number of exposures. Data are expressed as the mean ± SEM; * *p* < 0.05, ** *p* < 0.01, *** *p* < 0.001.

ICAM-1 was significantly increased following a 1× 16 psi exposure from the front (~172%), 2× 16 psi exposures from the side (~26%), 2× 13 psi exposures from the side (~32%), 4× 16 psi exposures from the side (~69%), and 4× 13 psi exposures from the side (~127%) (Figure 3).

### 2.3. Interferon-Gamma (IFN-γ)

**Figure 4:**
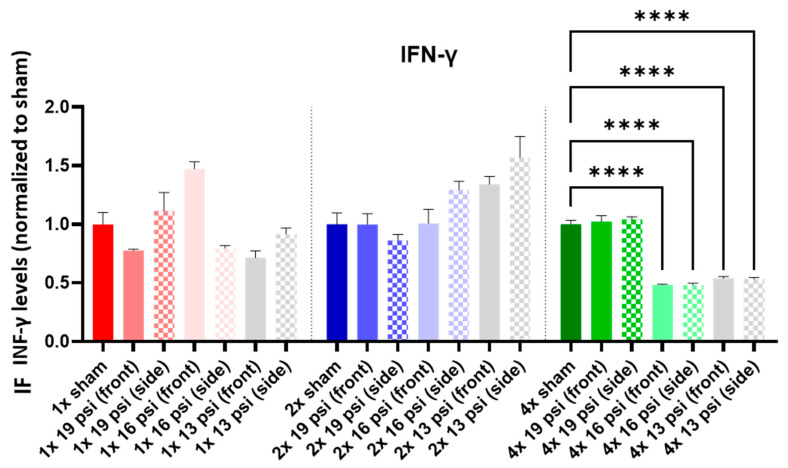
IFN-γ expression following repeated BW: IFN-γ was significantly decreased following four exposures to 16 psi (front and side) and 13 psi (front and side). Values are normalized to respective shams with the same number of exposures. Data are expressed as the mean ± SEM; **** *p* < 0.0001.

IFN-γ was significantly reduced following 4× 16 (~51%) and 13 psi (~46%) exposures (Figure 4).

### 2.4. Interleukins (ILs)

**Figure 5:**
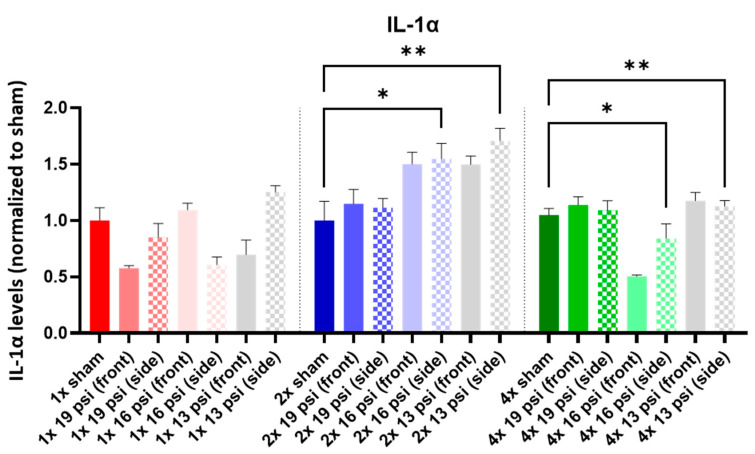
IL-1α expression following repeated BW: IL-1α was significantly increased following two exposures to 16 psi (side) and 13 psi (side), and after four exposures to 13 psi (side), and significantly decreased following four exposures to 16 psi (side). Values are normalized to respective shams with the same number of exposures. Data are expressed as the mean ± SEM; * *p* < 0.05, ** *p* < 0.01.

**Figure 6:**
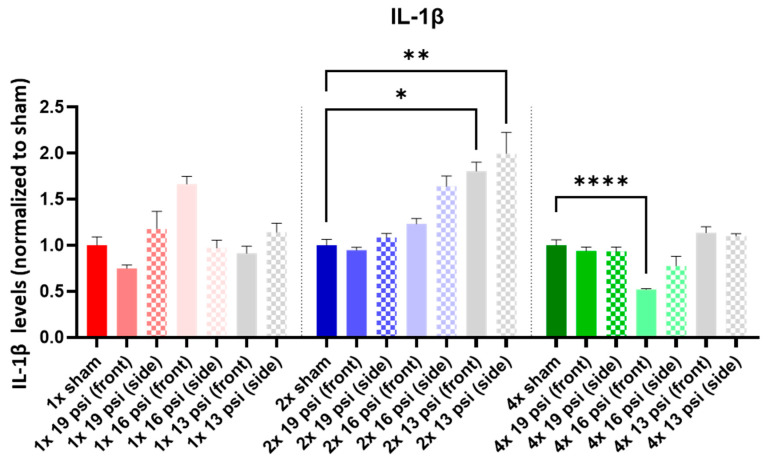
IL-1β expression following repeated BW: IL-1β was significantly increased following two exposures to 13 psi (front and side) and significantly decreased following four exposures to 16 psi (front). Values are normalized to respective shams with the same number of exposures. Data are expressed as the mean ± SEM; * *p* < 0.05, ** *p* < 0.01, **** *p* < 0.0001.

**Figure 7:**
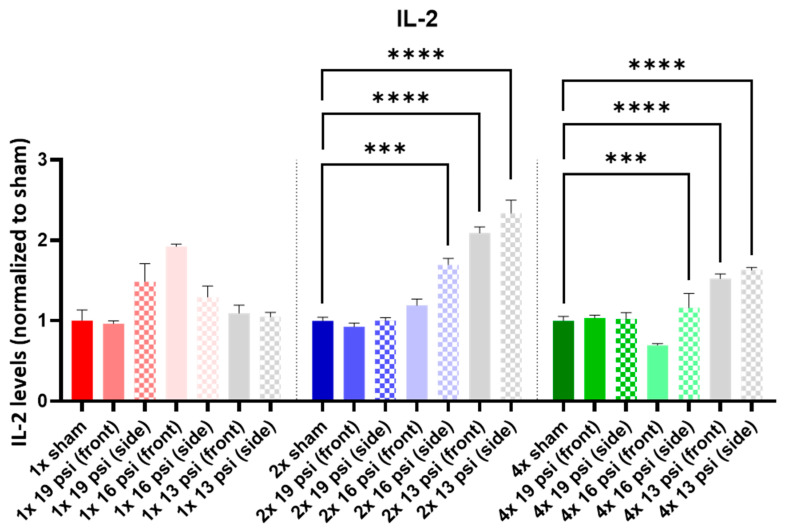
IL-2 expression following repeated BW: IL-2 was significantly increased following two exposures to 16 psi (side) and 13 psi (front and side), and following four exposures to 16 psi (side) and 13 psi (front and side). Values are normalized to respective shams with the same number of exposures. Data are expressed as the mean ± SEM; *** *p* < 0.001, **** *p* < 0.0001.

**Figure 8:**
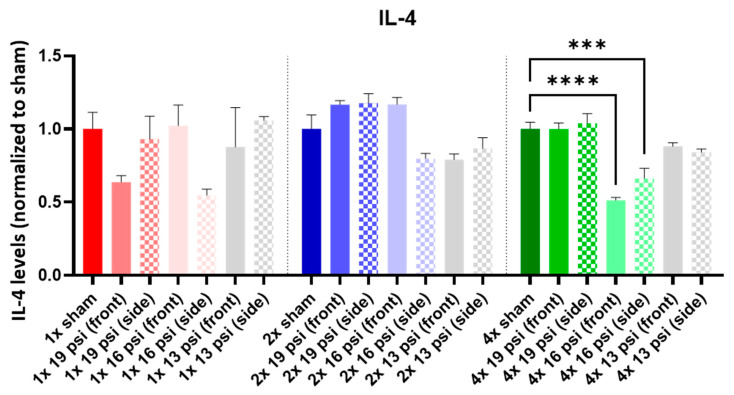
IL-4 expression following repeated BW: IL-4 was significantly decreased following four exposures to 16 psi (front and side). Values are normalized to respective shams with the same number of exposures. Data are expressed as the mean ± SEM; *** *p* < 0.001, **** *p* < 0.0001.

**Figure 9:**
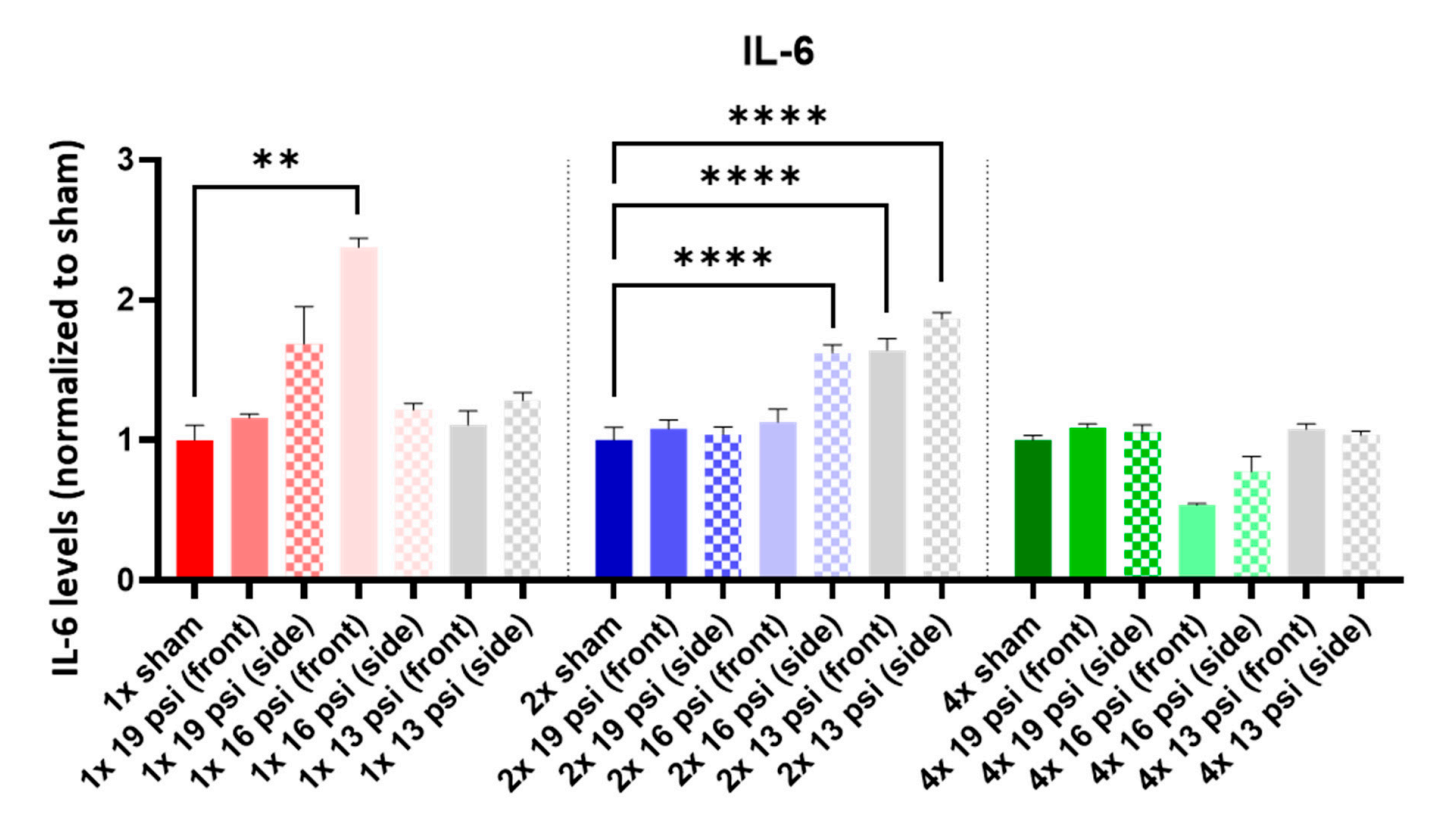
IL-6 expression following repeated BW: IL-6 was significantly increased following a single exposure to 16 psi (front) and two exposures to 16 psi (side) and 13 psi (front and side). Values are normalized to respective shams with the same number of exposures. Data are expressed as the mean ± SEM; ** *p* < 0.01, **** *p* < 0.0001.

**Figure 10:**
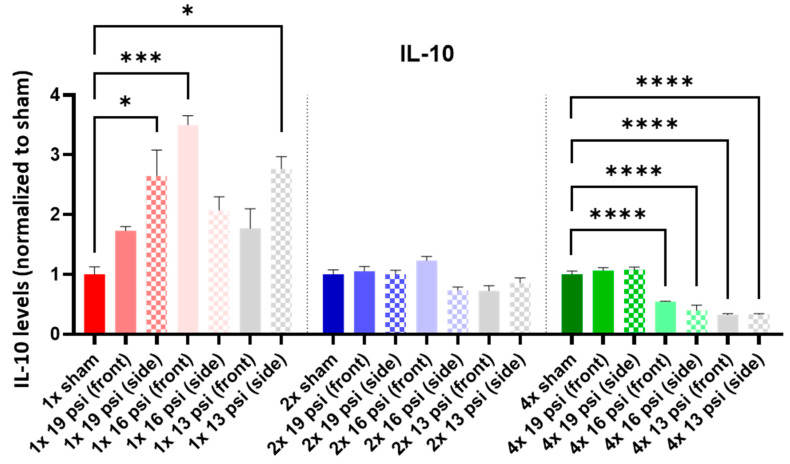
IL-10 expression following repeated BW: IL-10 was significantly increased following a single exposure to 19 psi (side), 16 psi (front), and 13 psi (side), and significantly decreased following four exposures to 16 psi (front and side) and 13 psi (front and side). Values are normalized to respective shams with the same number of exposures. Data are expressed as the mean ± SEM; * *p* < 0.05, *** *p* < 0.001, **** *p* < 0.0001.

**Figure 11:**
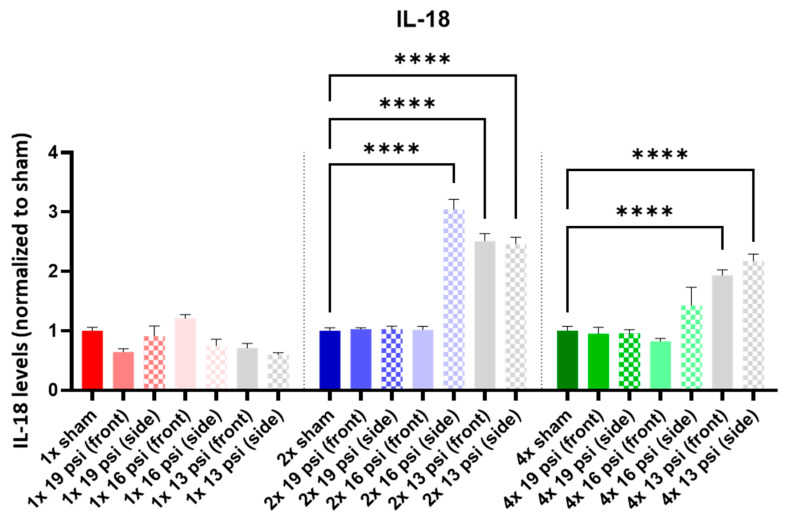
IL-18 expression following repeated BW: IL-18 was significantly increased following two exposures to 16 psi (side) and 13 psi (front and side), and following four exposures to 13 psi (front and side). Values are normalized to respective shams with the same number of exposures. Data are expressed as the mean ± SEM; **** *p* < 0.0001.

IL-1α was not altered following a one-time (1×) exposure at any of the tested intensities. It was significantly increased following 2× exposures to 16 psi and 13 psi from the side (~55% and ~71%, respectively) and 4× 13 psi exposures from the side (~13%). Conversely, IL-1α was significantly reduced following 4× 16 psi exposures from the side (~16%) (Figure 5). IL-1β was significantly increased following 2× 13 psi exposures from the front (~80%) and side (~100%) and significantly decreased following 4× 16 psi exposures from the front (~48%) (Figure 6). IL-2 was significantly increased following 2× 16 psi exposures from the side (~70%) and 2× 13 psi exposures from the front (~107%) and side (~140%). It was also significantly increased following 4× 16 psi exposures from the side (~16%) and 4× 13 psi exposures from the front (~50%) and side (~62%) (Figure 7). IL-4 was significantly reduced following 4×-16 psi exposures from the front (~49%) and side (~34%) (Figure 8). IL-6 was significantly increased following a 1× 16 psi exposure from the front (~138%) and following 2× 16 psi exposures from the side (~62%), 2× 13 psi exposures from the front (~64%), and 13 psi exposures from the side (~87%) (Figure 9). IL-10 was significantly increased following a 1× 19 psi exposure from the side (~164%), 1× 16 psi exposure from the front (~250%), and 1× 13 psi exposure from the side (~177%). It was significantly decreased following 4× 16 psi exposures from the front (~45%) and side (~60%) and 13 psi exposures from the front (~67%) and side (~67%) (Figure 10). IL-18 was significantly increased following 2× 16 psi exposures from the side (~205%) and 13 psi exposures from the front (~151%) and side (~146%). It was also increased following 4× 13 psi exposures from the front (~94%) and side (~117%) (Figure 11).

### 2.5. Tissue Inhibitor of Metalloproteinases (TIMP) Metallopeptidase Inhibitor 1 (TIMP1)

**Figure 12:**
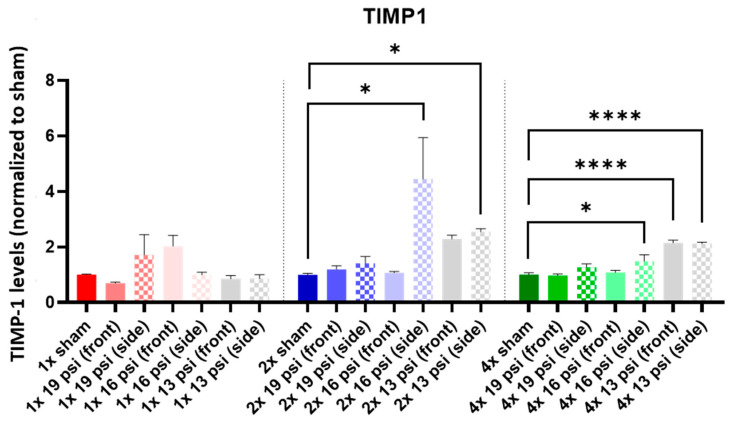
TIMP1 expression following repeated BW: TIMP1 was significantly increased following two exposures to 16 psi (side) and 13 psi (side), and following four exposures to 16 psi (side) and 13 psi (front and side). Values are normalized to respective shams with the same number of exposures. Data are expressed as the mean ± SEM; * *p* < 0.05, **** *p* < 0.0001.

TIMP1 was not significantly altered following a 1× BW exposure. It was significantly increased following 2× 16 psi exposures from the side (~345%) and 13 psi exposures from the side (~155%), as well as following 4× 16 psi exposures from the side (~49%) and 13 psi exposures from the front (~115%) and side (~113%) (Figure 12).

### 2.6. Tumor Necrosis Factor-Alpha (TNF-α)

**Figure 13:**
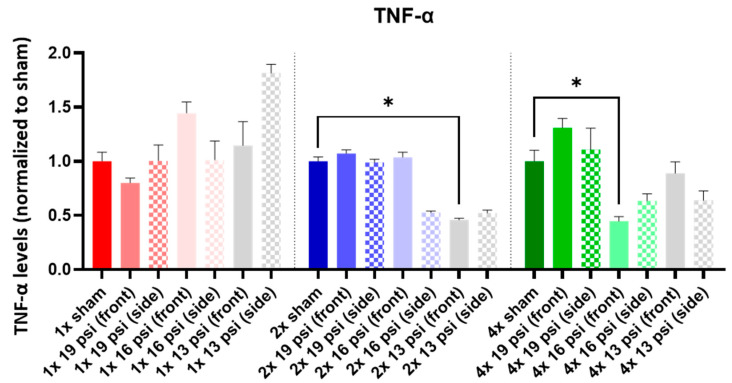
TNF-α expression following repeated BW: TNF-α was significantly decreased following two exposures to 13 psi (front) and four exposures to 16 psi (front). Values are normalized to respective shams with the same number of exposures. Data are expressed as the mean ± SEM. * *p* < 0.05.

TNF-α was not significantly altered following a single BW exposure. It was significantly reduced following 2× exposures to 13 psi from the front (~54%) and four exposures to 16 psi from the front (~55%) (Figure 13).

## 3. Discussion

There is ample evidence that BOP exposure is causally associated with neuropsychological disorders, but the underlying molecular mechanisms remain to be elucidated. A study of 275 Iraq and Afghanistan combat veterans found that blast severity was associated with diagnosis of PTSD and reported psychiatric and depressive symptoms [20,21]. Another study of military personnel from the United Kingdom deployed in Afghanistan found that PTSD symptoms were closely associated with IED detonation exposure compared with those who were not exposed to BOP [22]. In this study, we focused on the link between BW exposure and psychological disorders such as depression and PTSD, hypothesizing that neuroinflammation could be that molecular link. These psychological disorders are marked by various neuroinflammatory changes, and here we show that BOP exposure can be a source of disease-promoting neuroinflammation. Our group has reported alterations in mechanosensitive cation channel receptor, piezo 2, neurodegeneration-associated protein, TAR DNA-binding protein-43, and changes in neurovascular components associated with the blood–brain barrier (e.g., VEGF, claudin-4, occludin) with one, two, or four exposures at 13, 16, and 19 psi [23]. In addition, we have previously shown that BW leads to anxiety- and depression-like behavior at both acute and chronic timepoints [22]. Identical to previous studies, in this study we exposed rats to BW from different orientations (front- or side-facing), at different intensities (13 psi, 16 psi, 19 psi), and with different numbers of exposures (one, two, or four).

We found that high-intensity blasts caused the fewest inflammatory changes, with only CXCL3 and IL-10 increasing following a single 19 psi blast from the side. Following a single moderate front-facing blast (16 psi), CXCL2, intercellular adhesion molecule (ICAM)-1, IL-2, IL-6, and IL-10 were increased in the brain. Several pro-inflammatory cytokines were increased following multiple lower-intensity (13 psi) exposures, including IL-1a, IL-1b, and IL-6. We found that repeated low–moderate-intensity BW exposures led to inflammatory changes that were associated with both TBI and psychological stress.

The chemokine (C-X-C) motif ligands CXCL2 and CXCL3 are neutrophil chemoattractants that are involved in the inflammatory response [24,25]. In the current study, CXCL3 was increased in the brain following a single exposure to 19 psi from the side and 16 psi from the front, as well as following two exposures to 13 psi from the front or side (Figure 1), indicating that repeated exposures cause a heightened inflammatory state compared to that seen following a single exposure. However, CXCL2 has been shown to be increased in the brain and blood following brain injury, and it is associated with the onset of PTSD [24,26,27,28,29]. We found that CXCL2 was significantly decreased in the brain following four exposures to 16 psi from the front (Figure 2), indicating that it is unlikely that this level of BW exposure would cause a CXCL2-mediated injury response.

Intercellular adhesion molecule 1 (ICAM-1) is a transmembrane glycoprotein with key functions in inflammation and blood–brain barrier maintenance [12]. It is increased in the brain following impact injury and in the cerebrospinal fluid (CSF) of severe TBI patients [30,31]. Soluble ICAM-1 is increased in the blood of patients with depression, bipolar disorder, and dementia [12,32,33]. An ICAM-1-related link between brain injury and psychological stress was found when mice that were exposed to experimental impact mTBI and traumatic stress had increased ICAM-1 in the brain [34]. In our study, ICAM-1 was significantly increased in the brain following a single exposure to 16 psi from the front, two exposures to 16 or 13 psi from the side, and four exposures to 16 or 13 psi from the side (Figure 3). This is similar to the increase reported by Ojo et al., indicating that BW may cause an ICAM-1 response linked to psychological stress or depression [34].

Interferon (IFN)-γ is a cytokine that is involved in innate and adaptive immunity [35]. In a study of pediatric TBI, Ryan et al. found that IFN-γ was increased in mTBI patients relative to controls, but decreased in severe TBI patients relative to controls [36]. It was also found to be increased in the hippocampus of rats following a single 19 psi BW exposure [37]. However, we found that IFN-γ was not significantly altered following a single 19 psi BW exposure (Figure 4). One reason for this discrepancy is that Cho et al. measured IFN-γ levels in the hippocampus alone, while our brain homogenates were composed of the entire left cerebrum, so if there were region-specific alterations in this cytokine, they may have been masked by dissimilar responses in other regions. We did see a significant reduction in IFN-γ in the brain following four exposures to 13 and 16 psi from the front and side (Figure 4). IFN-γ levels have been found to be increased in patients with PTSD, generalized anxiety disorder (GAD), and major depressive disorder [38,39,40]. The decrease that we observed may indicate that blasts do not cause psychiatric-disorder-related IFN-γ alterations.

Interleukins are a family of cytokines that play pro- and anti-inflammatory roles in the activation and differentiation of immune cells [41]. In this study, we measured interleukin (IL)-1α, -1β, -2, -4, -6, -10, and -18. IL-α is a pro-inflammatory cytokine that is generated and released during acute phases of TBI [42], where it might exert a protective effect [43]. It was found to be increased in patients with GAD but decreased in the dorsolateral prefrontal cortex in subjects (or patients) with PTSD and depression [40,44]. We found that IL-1α was significantly increased in the brain following two exposures to 16 psi from the side and 13 psi from the side (Figure 5). It was also significantly increased following four exposures to 13 psi from the side, but decreased following four exposures to 16 psi from the side (Figure 5). It appeared that the increase in IL-1α that occurred after 2× 16 psi (side) was attenuated and levels became significantly lower following four exposures. IL-1α remained elevated in the brain after multiple exposures to 13 psi from the side, but the increase over shams became less pronounced after four exposures as compared to two exposures. This transient alteration may reflect a similarly transient impact on blast-induced psychological changes after repeated blasts. IL-1β is a pro-inflammatory cytokine that has been shown to be upregulated in the brain following brain injury [24,27,28] and in serum following blast TBI [45]. We found that IL-1β was significantly increased in the brain following two exposures to 13 psi from the front or side, but significantly decreased following four exposures to 16 psi from the front (Figure 6). IL-1β was significantly increased in the sera of patients with depressive disorder, showing a positive correlation with depression’s severity [46]. It was also increased in PTSD patients [16]. The increase in IL-1β that we observed in the two-exposure groups may indicate a transient depression/PTSD-like inflammatory state that is dampened following further blast exposures.

IL-2 is another pro-inflammatory cytokine that has been found to be increased in the sera of rats following blast TBI [45]. Although that study was performed using TNT detonations rather than an advanced blast simulator, we found similar increases in IL-2 following blasts, with significant increases in the brain following multiple (i.e., two or four) exposures to 16 psi from the side and 13 psi from the front and side (Figure 7). This cytokine has been found to be increased in the plasma of mTBI patients within 24 h of injury, and elevated plasma IL-2 was associated with more severe post-concussive symptoms one week post-injury [47]. It was also increased in GAD patients [40]. The increases in IL-2 that we observed might predict more post-concussion symptoms after the acute phase and may predict an associated anxiety-like state.

IL-4 is an anti-inflammatory cytokine. Jiang, et al. reported that CCI did not alter IL-4 levels in the brain; however, intranasal treatment of CCI-injured mice with IL-4 boosted their neurological recovery, indicating a protective function following TBI [48,49]. We found that IL-4 was significantly decreased in the brain following four exposures to 16 psi from the front and side (Figure 8). This decrease indicates that the protective, anti-inflammatory functions of IL-4 were likely diminished in these animals. IL-6 is a pro-inflammatory cytokine that has been found to be increased in the cortex following CCI [27,28] and increased in serum following experimental bTBI [45]. In TBI patients, it was increased in both the CSF and serum [36,43,47]. We measured a significant increase in IL-6 in the brain following a single exposure to 16 psi from the front, and following two exposures to 16 psi from the side and 13 psi from the front and side (Figure 9).

IL-10 is an anti-inflammatory cytokine and is decreased in mTBI patients compared with controls [36]. We found a significant increase in IL-10 following single exposures to 19 psi from the side, 16 psi from the front, and 13 psi from the side, but a significant decrease following four exposures to 16 and 13 psi from the front and side (Figure 10). IL-18 is a pro-inflammatory cytokine that is associated with TBI’s pathogenesis [50,51,52,53]. It is increased in the CSF of TBI patients, and levels of circulating IL-18 are correlated with chronic TBI patients’ cognitive impairment and disability severity [54,55]. We found that IL-18 was significantly increased in the brain following two or four exposures to 16 psi from the side and 13 psi from the front and side (Figure 11). Changes in the IL markers have been commonly associated with neurodegenerative and neuropsychiatric disorders [46,47,56,57].

TIMP1 is a regulator of matrix metalloproteinases and, therefore, is important in extracellular matrix maintenance. It has been found to be increased following injury in both TBI animal models [58,59] and TBI patients [60,61]. Treatment of mice with recombinant TIMP1 following experimental TBI exerts a neuroprotective function by ameliorating blood–brain barrier disruption [62]. Therefore, the increase in TIMP1 observed following TBI is likely a compensatory or repair response. Similarly, we observed a significant increase in TIMP1 in the brain following two exposures to 16 psi or 13 psi from the side, and following four exposures to 16 psi from the side and 13 psi from the front and side (Figure 12). Dysregulation of TIMP1 expression is hypothesized to be a basis for abnormal cognitive abilities, and its upregulation may be responsible for the development of major depressive disorder [63]. The upregulation that we observed could therefore contribute to development of depressive symptoms while repairing BBB disruption.

TNF-α is a pro-inflammatory cytokine that is elevated in the CSF of severe TBI patients [31]. In experimental TBI, it was similarly increased in the cortex following impact injury and in the serum following detonation blast injury [24,28,45]. However, it was significantly reduced in pediatric mTBI patients vs. controls at baseline [36]. We found that TNF-α was significantly reduced following two exposures to 13 psi from the front and after four exposures to 16 psi from the front (Figure 13). The rats in our study were 8–9 weeks old, which would correspond to an adolescent age range [64], so the reduction in TNF-α that we observed might best reflect the findings in adolescents by Ryan et al. [36]. TNF-α is increased in PTSD patients and in chronic-stress-induced depressive-like mice [16,39,56]. Because TNF-α was reduced following blast exposure in our cohort of rats, we did not find an obvious TNF-α-mediated link between blast TBI and psychological alterations.

Overall, we can speculate that these changes are inconsistent across several cytokines and chemokines because of the differences in the intensity of pressure. These differences may have unique consequences for the brain, systemic inflammation, lung trauma, and effects on other organs that may have secondary consequences to these changes [65,66]. For example, in our previous study, we observed varied levels of lung trauma among the groups, with four exposures to 19 psi having the highest degree of lung injury and 1× 13 psi having the lowest degree of lung injury. Furthermore, when four exposures from the front and side were compared, we observed significant levels of lung injury in the side exposure group [65]. These factors could contribute to differential changes across the groups of exposure and frequency. In addition, it is important to evaluate these changes over time to fully understand the extent of the changes that may be dependent on the frequency and intensity of the exposures. These limitations of the current study need to investigated in future studies, along with histopathological studies. Additional limitations include the lack of understanding of sex differences, the use of a small animal model, and assessments of whole-brain homogenates that may have compromised the understanding of regional differences in the brain.

## 4. Materials and Methods

### 4.1. Animals

All animal experiments were conducted in accordance with the Animal Welfare Act and other federal statutes and regulations relating to animals and experiments involving animals, and they adhered to the principles stated in the *Guide for the Care and Use of Laboratory Animals* (NRC Publication 2011 edition) using a protocol approved by the Institutional Animal Care and Use Committee. Male Sprague Dawley rats, 8–9 weeks old (n = 6 per group) and weighing ~275 g (Charles River Laboratories, Wilmington, MA, USA), were housed at 20–22 °C (12 h light/dark cycle) with free access to food and water ad libitum.

### 4.2. BW Exposure

**Figure 14:**
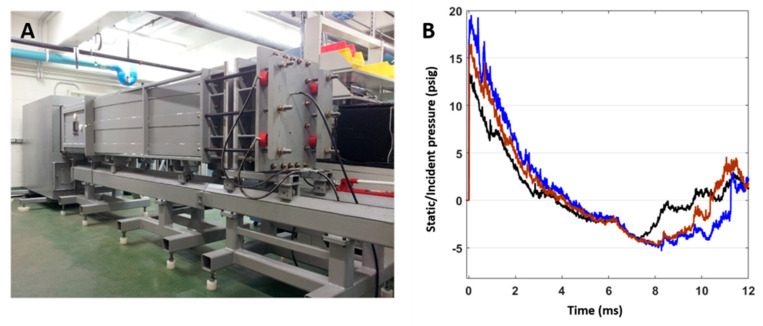
(**A**) The advanced blast simulator (ABS) located at the Walter Reed Army Institute of Research (WRAIR), used to produce the experimental blasts. (**B**) Pressure profiles generated using the ABS, which have both positive and negative phases and mimic “free-field” blasts for the 13 (black), 16 (brown), and 19 psi (blue) treatment groups.

Rats were anesthetized with 4% isoflurane and subjected to survivable BW exposures using an ABS located at the Walter Reed Army Institute of Research (WRAIR). The ABS consists of a 0.5 ft long compression chamber that is separated from a 21 ft long transition/expansion test section (Figure 14). The anesthetized rat was secured in the test section in a longitudinal (head-on; on-axis) or transverse (side-on; off-axis) orientation to the direction of shockwave propagation. The compression chamber was pressurized with room air, causing membranes to rupture at a pressure dependent upon the thickness of the specific membrane sheet separating the two chambers, yielding a supersonic blast wave that impacted the experimental subject in the test section. To yield a range of mild-to-moderate TBI in rats in these experiments, Valmex^®^ membranes (Mehler Texnologies, Martinsville, VA, USA) were used to yield peak positive static pressures of 13 (impulse: 17.27 ± 0.51 psi*ms), 16 (impulse: 23.99 ± 0.51 psi*ms), and 19 psi (impulse: 29.87 ± 0.51 psi*ms), with a positive-phase duration of 4–5 ms, and with negative peak static pressures of 3.96 ± 0.11 psi, 4.61 ± 0.18 psi, and 4.76 ± 0.19 psi respectively. Animals (n = 6 per group) were exposed to a daily blast (one per day) of 13, 16, or 19 psi either once (1×), twice (2×), or four times (4×), from the front or from the side; repeated blast exposures were separated by 24 h [23]. All sham animals were subjected to isoflurane anesthesia, loading in the shock tube, and recovery procedures as described above, but they were not exposed to BW (BW). At 24 h following the final BW exposure, the animals were euthanatized, and whole-hemisphere brain tissue was flash-frozen on dry ice until further analysis.

### 4.3. Protein Extraction

After euthanasia, the right cerebrum was homogenized in 5% *w*/*v* T-PER Tissue Protein Extraction Reagent (Thermo Fisher, New York, NY, USA) with a 1% protease/phosphatase inhibitor cocktail (Sigma-Aldrich, St. Louis, MO, USA). The homogenate was centrifuged at 5000× *g* for 5 min at 4 °C. The supernatant, containing the soluble protein fraction, was collected and stored at −80 °C until use for multiplex ELISA.

### 4.4. Multiplex ELISA

A bead-based multiplex cytokine kit (LXSARM-14, R&D Systems, Minneapolis, MN, USA) was used to measure brain levels of CXC2, CXCL3, ICAM-1, IFN-γ, IL-1α, IL-1β, IL-2, IL-4, IL-6, IL-10, IL-18, TIMP1, and TNF-α. All samples were run in triplicate, and the assay was run according to the manufacturer’s instructions. Plates were read using xPonent 3.1 software on a MAGPIX system (Luminex Corp, Austin, TX, USA).

### 4.5. Statistical Analysis

All results were normalized to respective shams, such that 1× experimental groups were compared to 1× shams and 4× experimental groups were compared to 4× shams. The Shapiro–Wilk test of normality was used to determine whether the datasets had a normal distribution. In normal datasets, ordinary one-way analysis of variance (ANOVA) was performed (with Tukey’s multiple comparison test); otherwise, the non-parametric Kruskal–Wallis test was performed (with Dunn’s multiple comparison test). A significance level of *p* < 0.05 was considered statistically significant (* *p* < 0.05, ** *p* < 0.01, *** *p* < 0.001, **** *p* < 0.0001.) Unless otherwise specified, all data are expressed as the mean ± SEM.

## 5. Conclusions

Overall, we found that blast exposure caused many neuroinflammatory changes in the brain that are similar to those found in depression, PTSD, and other psychiatric disorders. These negative psychological health outcomes are prevalent in veterans and soldiers, and finding the underlying molecular causes of these disorders is necessary and important for targeted treatments. We identified several inflammatory markers that seemed to present a link between military-relevant blast exposure and disease-related neuroinflammation. We predict that targeting of this neuroinflammation may be a viable treatment option for the prevention of blast-related behavioral outcomes following blast exposure, and for determining whether treatment of the neuroinflammation may prevent these negative behavioral outcomes.

## 6. Disclaimer

This material has been reviewed by the Walter Reed Army Institute of Research. There is no objection to its presentation and/or publication. The opinions or assertions contained herein are the private views of the authors and are not to be construed as official, or as reflecting the true views of the Department of the Army or the Department of Defense. This research was conducted under an IACUC-approved animal use protocol in an AAALAC-International-accredited facility with a Public Health Services Animal Welfare Assurance, and in compliance with the Animal Welfare Act and other federal statutes and regulations relating to laboratory animals.

## Figures and Tables

**Table 1:** Summary of neuroinflammatory responses to BW; up arrows indicate a significant increase, and down arrows indicate a significant decrease. Red = 1×, green = 2×, black = 4×.

	19 psi (Front)	19 psi (Side)	16 psi (Front)	16 psi (Side)	13 psi (Front)	13 psi (Side)
**CXCL3**		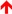	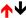		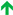	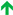
**CXCL2**			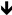			
**ICAM-1**			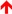	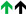		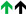
**IFN-γ**			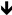	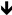	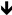	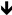
**IL-1α**				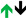		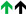
**IL-1β**					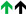	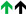
**IL-2**			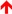	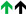	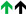	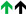
**IL-4**			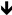	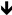		
**IL-6**			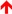	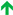	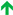	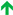
**IL-10**		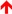	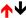	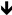	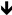	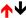
**IL-18**				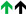	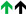	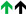
**TIMP1**				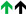	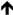	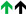
**TNF-α**			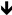		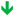	

## Data Availability

Raw data is available upon request to authors.

## References

[1] Traumatic Brain Injury Center of Excellence (TBICoR) (2018). DoD Numbers for Traumatic Brain Injury: Worldwide Totals. https://health.mil/Military-Health-Topics/Centers-of-Excellence/Traumatic-Brain-Injury-Center-of-Excellence/DOD-TBI-Worldwide-Numbers.

[2] Hoge C.W., McGurk D., Thomas J.L., Cox A.L., Engel C.C., Castro C.A. (2008). Mild Traumatic Brain Injury in U.S. Soldiers Returning from Iraq. N. Engl. J. Med..

[3] Walz R., Schwarzbold M., Diaz A., Martins E.T., Rufino A., Amante L.N., Thais M.E., Quevedo J., Hohl A., Linhares M.N. (2008). Psychiatric disorders and traumatic brain injury. Neuropsychiatr. Dis. Treat..

[4] Morissette S.B., Woodward M., Kimbrel N.A., Meyer E.C., Kruse M.I., Dolan S., Gulliver S.B. (2011). Deployment-Related TBI, Persistent Postconcussive Symptoms, PTSD, and Depression in OEF/OIF Veterans. Rehabil. Psychol..

[5] Laughter S., Khan M., Banaag A., Madsen C., Koehlmoos T.P. (2021). Prevalence of Polytrauma Clinical Triad Among Active Duty Service Members. Mil. Med..

[6] Gill J., Motamedi V., Osier N., Dell K., Arcurio L., Carr W., Walker P., Ahlers S., LoPresti M., Yarnell A. (2017). Moderate blast exposure results in increased IL-6 and TNFα in peripheral blood. Brain Behav. Immun..

[7] Li Y., Yang Z., Liu B., Valdez C., Chavko M., Cancio L.C. (2019). Low-Level Primary Blast Induces Neuroinflammation and Neurodegeneration in Rats. Mil. Med..

[8] Sajja V.S.S.S., Tenn C., McLaws L.J., VandeVord P.J. (2012). A temporal evaluation of cytokines in rats after blast exposure. Biomed. Sci. Instrum..

[9] Toklu H.Z., Yang Z., Oktay S., Sakarya Y., Kirichenko N., Matheny M.K., Muller-Delp J., Strang K., Scarpace P.J., Wang K.K. (2018). Overpressure blast injury-induced oxidative stress and neuroinflammation response in rat frontal cortex and cerebellum. Behav. Brain Res..

[10] Sosa M.A.G., De Gasperi R., Pryor D., Garcia G.S.P., Perez G.M., Abutarboush R., Kawoos U., Hogg S., Ache B., Janssen W.G. (2021). Low-level blast exposure induces chronic vascular remodeling, perivascular astrocytic degeneration and vascular-associated neuroinflammation. Acta Neuropathol. Commun..

[11] Pandey G.N., Rizavi H.S., Bhaumik R., Zhang H. (2021). Chemokines gene expression in the prefrontal cortex of depressed suicide victims and normal control subjects. Brain Behav. Immun..

[12] Müller N. (2019). The Role of Intercellular Adhesion Molecule-1 in the Pathogenesis of Psychiatric Disorders. Front. Pharmacol..

[13] Dowlati Y., Herrmann N., Swardfager W., Liu H., Sham L., Reim E.K., Lanctôt K.L. (2010). A Meta-Analysis of Cytokines in Major Depression. Biol. Psychiatry.

[14] Himmerich H., Patsalos O., Lichtblau N., Ibrahim M.A.A., Dalton B. (2019). Cytokine Research in Depression: Principles, Challenges, and Open Questions. Front. Psychiatry.

[15] Smid G.E., van Zuiden M., Geuze E., Kavelaars A., Heijnen C.J., Vermetten E. (2015). Cytokine production as a putative biological mechanism underlying stress sensitization in high combat exposed soldiers. Psychoneuroendocrinology.

[16] Hori H., Kim Y. (2019). Inflammation and post-traumatic stress disorder. Psychiatry Clin. Neurosci..

[17] Mehta D., Voisey J., Bruenig D., Harvey W., Morris C.P., Lawford B., Young R.M. (2018). Transcriptome analysis reveals novel genes and immune networks dysregulated in veterans with PTSD. Brain Behav. Immun..

[18] Kanefsky R., Motamedi V., Mithani S., Mysliwiec V., Gill J.M., Pattinson C.L. (2019). Mild traumatic brain injuries with loss of consciousness are associated with increased inflammation and pain in military personnel. Psychiatry Res..

[19] Heyburn L., Abutarboush R., Goodrich S., Urioste R., Batuure A., Wheel J., Wilder D.M., Arun P., Ahlers S.T., Long J.B. (2021). Repeated Low-Level Blast Acutely Alters Brain Cytokines, Neurovascular Proteins, Mechanotransduction, and Neurodegenerative Markers in a Rat Model. Front. Cell. Neurosci..

[20] Martindale S.L., Ord A.S., Rule L.G., Rowland J.A. (2021). Effects of blast exposure on psychiatric and health symptoms in combat veterans. J. Psychiatr. Res..

[21] Jones N., Thandi G., Fear N.T., Wessely S., Greenberg N. (2014). The psychological effects of improvised explosive devices (IEDs) on UK military personnel in Afghanistan. Occup. Environ. Med..

[22] Arun P., Wilder D.M., Eken O., Urioste R., Batuure A., Sajja S., Van Albert S., Wang Y., Gist I.D., Long J.B. (2020). Long-Term Effects of Blast Exposure: A Functional Study in Rats Using an Advanced Blast Simulator. J. Neurotrauma.

[23] Heyburn L., Abutarboush R., Goodrich S., Urioste R., Batuure A., Statz J., Wilder D., Ahlers S.T., Long J.B., Sajja V.S. (2019). Repeated Low-Level BW Leads to Endovascular Disruption and Alterations in TDP-43 and Piezo2 in a Rat Model of Blast TBI. Front. Neurol..

[24] Szmydynger-Chodobska J., Strazielle N., Zink B.J., Ghersi-Egea J.-F., Chodobski A. (2009). The Role of the Choroid Plexus in Neutrophil Invasion after Traumatic Brain Injury. J. Cereb. Blood Flow Metab..

[25] Ermakov E.A., Mednova I.A., Boiko A.S., Buneva V.N. (2022). Chemokine Dysregulation and Neuroinflammation in Schizophrenia: A Systematic Review. Int. J. Mol. Sci..

[26] Rhodes J.K., Sharkey J., Andrews P.J., Loane D.J., Kumar A., Su E., Bell M., Taka E., Mazzio E.A., Goodman C.B. (2009). The Temporal Expression, Cellular Localization, and Inhibition of the Chemokines MIP-2 and MCP-1 after Traumatic Brain Injury in the Rat. J. Neurotrauma.

[27] Chen C.-C., Hung T.-H., Wang Y.-H., Lin C.-W., Wang P.-Y., Lee C.-Y., Chen S.-F. (2012). Wogonin Improves Histological and Functional Outcomes, and Reduces Activation of TLR4/NF-κB Signaling after Experimental Traumatic Brain Injury. PLoS ONE.

[28] Zhao X., Liu S., Yang X., Liu Y., Liu G., Fan K., Ma J. (2021). Cathepsin C aggravates neuroinflammation via promoting production of CCL2 and CXCL2 in glial cells and neurons in a cryogenic brain lesion. Neurochem. Int..

[29] Zhang L., Hu X.-Z., Li X., Chen Z., Benedek D.M., Fullerton C.S., Wynn G., Naifeh J.A., Wu H., Benfer N. (2020). Potential chemokine biomarkers associated with PTSD onset, risk and resilience as well as stress responses in US military service members. Transl. Psychiatry.

[30] Rancan M., Otto V.I., Hans V.H., Gerlach I., Jork R., Trentz O., Kossmann T., Morganti-Kossmann M.C. (2001). Upregulation of ICAM-1 and MCP-1 but not of MIP-2 and sensorimotor deficit in response to traumatic axonal injury in rats. J. Neurosci. Res..

[31] Otto V.I., Heinzel-Pleines U.E., Gloor S.M., Trentz O., Kossmann T., Morganti-Kossmann M.C. (2000). sICAM-1 and TNF-α induce MIP-2 with distinct kinetics in astrocytes and brain microvascular endothelial cells. J. Neurosci. Res..

[32] Yang X., Evans R.W., George C.J., Matthews K.A., Kovacs M. (2022). Adiposity and Smoking Mediate the Relationship Between Depression History and Inflammation Among Young Adults. Int. J. Behav. Med..

[33] Liu X., Huang J., Jiang Y., Cao Z., Wu M., Sun R., Chen Z., Yu P., Ma J., Chen Y. (2022). IL-6 and IL-8 are likely associated with psychological status in treatment naïve general population. J. Affect. Disord..

[34] Ojo J.O., Greenberg M.B., Leary P., Mouzon B., Bachmeier C., Mullan M., Diamond D.M., Crawford F. (2014). Neurobehavioral, neuropathological and biochemical profiles in a novel mouse model of co-morbid post-traumatic stress disorder and mild traumatic brain injury. Front. Behav. Neurosci..

[35] Burke J.D., Young H.A. (2020). IFN-γ: A cytokine at the right time, is in the right place. Semin. Immunol..

[36] Ryan E., Kelly L., Stacey C., Huggard D., Duff E., McCollum D., Leonard A., Boran G., Doherty D.R., Bolger T. (2022). Mild-to-severe traumatic brain injury in children: Altered cytokines reflect severity. J. Neuroinflammation.

[37] Cho H., Sajja V., VandeVord P., Lee Y. (2013). Blast induces oxidative stress, inflammation, neuronal loss and subsequent short-term memory impairment in rats RSS Download PDF. Neuroscience.

[38] Chen S., Zhang Y., Yuan Y. (2021). The Combination of Serum BDNF, Cortisol and IFN-Gamma Can Assist the Diagnosis of Major Depressive Disorder. Neuropsychiatr. Dis. Treat..

[39] Yang J.-J., Jiang W. (2020). Immune biomarkers alterations in post-traumatic stress disorder: A systematic review and meta-analysis. J. Affect. Disord..

[40] Tang Z., Ye G., Chen X., Pan M., Fu J., Fu T., Liu Q., Gao Z., Baldwin D.S., Hou R. (2017). Peripheral proinflammatory cytokines in Chinese patients with generalised anxiety disorder. J. Affect. Disord..

[41] Vaillant A.A.J., Qurie A. (2022). Interleukin.

[42] Thome J.G., Reeder E.L., Collins S.M., Gopalan P., Robson M.J. (2020). Contributions of Interleukin-1 Receptor Signaling in Traumatic Brain Injury. Front. Behav. Neurosci..

[43] Abboud A., Mi Q., Puccio A., Okonkwo D., Buliga M., Constantine G., Vodovotz Y. (2016). Inflammation Following Traumatic Brain Injury in Humans: Insights from Data-Driven and Mechanistic Models into Survival and Death. Front. Pharmacol..

[44] Morrison F.G., Miller M.W., Wolf E.J., Logue M.W., Maniates H., Kwasnik D., Cherry J.D., Svirsky S., Restaino A., Hildebrandt A. (2019). Reduced interleukin 1A gene expression in the dorsolateral prefrontal cortex of individuals with PTSD and depression. Neurosci. Lett..

[45] Ma J., Wang J., Cheng J., Xiao W., Fan K., Gu J., Yu B., Yin G., Wu J., Ren J. (2017). Impacts of Blast-Induced Traumatic Brain Injury on Expressions of Hepatic Cytochrome P450 1A2, 2B1, 2D1, and 3A2 in Rats. Cell. Mol. Neurobiol..

[46] Ogłodek E. (2022). Changes in the Serum Levels of Cytokines: IL-1β, IL-4, IL-8 and IL-10 in Depression with and without Posttraumatic Stress Disorder. Brain Sci..

[47] Vedantam A., Brennan J., Levin H.S., McCarthy J.J., Dash P.K., Redell J.B., Yamal J.-M., Robertson C.S. (2021). Early versus Late Profiles of Inflammatory Cytokines after Mild Traumatic Brain Injury and Their Association with Neuropsychological Outcomes. J. Neurotrauma.

[48] Jiang Q., Wei D., He X., Gan C., Long X., Zhang H. (2021). Phillyrin Prevents Neuroinflammation-Induced Blood–Brain Barrier Damage Following Traumatic Brain Injury via Altering Microglial Polarization. Front. Pharmacol..

[49] Pu H., Zheng X., Jiang X., Mu H., Xu F., Zhu W., Ye Q., Jizhang Y., Hitchens T.K., Shi Y. (2021). Interleukin-4 improves white matter integrity and functional recovery after murine traumatic brain injury via oligodendroglial PPARγ. J. Cereb. Blood Flow Metab..

[50] Marsland A.L., Walsh C., Lockwood K., John-Henderson N.A. (2017). The effects of acute psychological stress on circulating and stimulated inflammatory markers: A systematic review and meta-analysis. Brain Behav. Immun..

[51] Buspavanich P., Adli M., Himmerich H., Berger M., Busche M., Schlattmann P., Bopp S., Bschor T., Richter C., Steinacher B. (2021). Faster speed of onset of the depressive episode is associated with lower cytokine serum levels (IL-2, -4, -6, -10, TNF-α and IFN-γ) in patients with major depression. J. Psychiatr. Res..

[52] Kiraly D.D., Horn S.R., Van Dam N.T., Costi S., Schwartz J., Kim-Schulze S., Patel M., Hodes G.E., Russo S.J., Merad M. (2017). Altered peripheral immune profiles in treatment-resistant depression: Response to ketamine and prediction of treatment outcome. Transl. Psychiatry.

[53] Freeman L.C., Ting J.P.-Y. (2015). The pathogenic role of the inflammasome in neurodegenerative diseases. J. Neurochem..

[54] Kerr N., Lee S.W., Perez-Barcena J., Crespi C., Ibañez J., Bullock M.R., Dietrich W.D., Keane R.W., Vaccari J.P.D.R. (2018). Inflammasome proteins as biomarkers of traumatic brain injury. PLoS ONE.

[55] Ciaramella A., Della Vedova C., Salani F., Viganotti M., D’Ippolito M., Caltagirone C., Formisano R., Sabatini U., Bossù P. (2014). Increased Levels of Serum IL-18 Are Associated with the Long-Term Outcome of Severe Traumatic Brain Injury. Neuroimmunomodulation.

[56] Song A.-Q., Gao B., Fan J.-J., Zhu Y.-J., Zhou J., Wang Y.-L., Xu L.-Z., Wu W.-N. (2020). NLRP1 inflammasome contributes to chronic stress-induced depressive-like behaviors in mice. J. Neuroinflammation.

[57] Ogłodek E.A. (2017). The role of PON-1, GR, IL-18, and OxLDL in depression with and without posttraumatic stress disorder. Pharmacol. Rep..

[58] Russell K.L., Berman N.E., Levant B. (2013). Low brain DHA content worsens sensorimotor outcomes after TBI and decreases TBI-induced Timp1 expression in juvenile rats. Prostaglandins Leukot. Essent. Fat. Acids.

[59] Foerstner P., Rehman R., Anastasiadou S., Haffner-Luntzer M., Sinske D., Ignatius A., Roselli F., Knoell B. (2018). Neuroinflammation after Traumatic Brain Injury Is Enhanced in Activating Transcription Factor 3 Mutant Mice. J. Neurotrauma.

[60] Lorente L. (2015). New Prognostic Biomarkers in Patients With Traumatic Brain Injury. Arch. Trauma Res..

[61] Wang Z., Zheng K., Zheng P., Fan W., Li C., Liu H., Shan X. (2013). Matrix metalloproteinases and their tissue inhibitors in serum and cerebrospinal fluid of patients with moderate and severe traumatic brain injury. Neurol. India.

[62] Tang J., Kang Y., Huang L., Wu L., Peng Y. (2020). TIMP1 preserves the blood–brain barrier through interacting with CD63/integrin β1 complex and regulating downstream FAK/RhoA signaling. Acta Pharm. Sin. B.

[63] Wei Y., Qi K., Yu Y., Lu W., Xu W., Yang C., Lin Y. (2021). Analysis of Differentially Expressed Genes in the Dentate Gyrus and Anterior Cingulate Cortex in a Mouse Model of Depression. BioMed Res. Int..

[64] Sengupta P. (2013). The Laboratory Rat: Relating Its Age With Human’s. Int. J. Prev. Med..

[65] Sajja V.S., Statz J.K., Walker L.P.B., Gist I.D., Wilder D.M., Ahlers S.T., Long J.B. (2020). Pulmonary injury risk curves and behavioral changes from blast overpressure exposures of varying frequency and intensity in rats. Sci Rep..

[66] Bergmann-Leitner E.S., Bobrov A.G., Bolton J.S., Rouse M.D., Heyburn L., Pavlovic R., Garry B.I., Alamneh Y., Long J., Swierczewski B. (2022). Blast Waves Cause Immune System Dysfunction and Transient Bone Marrow Failure in a Mouse Model. Front. Bioeng. Biotechnol..

